# Sex effect on the fatty acid profile and chemical composition of meat from beef cattle fed a whole shelled corn diet

**DOI:** 10.5194/aab-66-51-2023

**Published:** 2023-01-31

**Authors:** Elizângela O. C. Santana, Robério R. Silva, Julliana I. Simionato, Geraldo Trindade Júnior, Túlio O. J. D'A. Lins, Gabriel D. da Costa, Bruna M. A. de C. Mesquita, Henry D. R. Alba, Gleidson G. P. de Carvalho

**Affiliations:** 1 Department of Animal Science, Universidade Estadual do Sudoeste da Bahia, Itapetinga 45.700-000, Brazil; 2 Department of Animal Science, Universidade Tecnológica Federal do Paraná, Londrina 86.036-370, Brazil; 3 Department of Animal Science, Universidade Federal de Minas Gerais, Montes Claros 39.404-547, Brazil; 4 Department of Animal Science, Universidade Federal da Bahia, Salvador 40.170-110, Brazil

## Abstract

This study aimed to evaluate the effect of sex on the chemical composition, fatty acid profile, and nutritional indexes of the *Longissimus lumborum* muscle of feedlot cattle fed a whole shelled corn diet. A total of 40 Nellore cattle were used, of which 20 animals were uncastrated bulls and 20 were heifers, with an average body weight of 411 ± 20.82 and 274 ± 17.80 kg, respectively. The diet was composed of 85 % whole shelled corn and 15 % protein–vitamin–mineral supplement. The chemical composition, fatty acid profile, and the nutritional value of the meat of the animals were evaluated. The chemical composition of the *Longissimus lumborum* muscle was similar among sexes (p>0.05). Differences were observed in the saturated (17 : 0, 18 : 0, and 21 : 0) and monounsaturated (16 : 1 *cis*-9, 17 : 1 *cis*-10, 18 : 1 *cis*-9, and 18 : 1 *trans*-7) fatty acid composition between sexes (p<0.05), and the polyunsaturated fatty acid (18 : 2 *cis*-9 and *cis*-12, 18:2 *cis*-9 and *trans*-11, 20 : 3 *n*-6, and 22 : 6 *n*-3) content also showed sex differences (p<0.05). The total number of saturated and polyunsaturated fatty acids was higher in bulls, and the total number of monounsaturated fatty acids was higher in heifers (p<0.05). Indicators of nutritional quality, such as the *n*-6 : *n*-3 ratio, thrombogenicity index, and Δ-9-desaturase C14, Δ-9-desaturase C16, and Δ-9-desaturase C18 indexes, were different between the sexes (p<0.05). In conclusion, sex was not found to influence chemical composition but did affect the fatty acid profile and nutritional indexes in the meat of feedlot cattle fed a whole shelled corn diet.

## 1 Introduction

Meat from cattle has been associated with the appearance of cardiovascular disease in humans due to its particular fat composition. Changing the fatty acid (FA) profile of beef to achieve a lower proportion of saturated fatty acids (SFAs) is an important way to produce healthier meat for the consumer (Ladeira et al., 2014). According to Scollan et al. (2014), rumen lipolysis and biohydrogenation are processes that act as limiting factors in improving the lipid profile of meat. Beef cattle meat is also composed of biohydrogenation products and unsaturated fatty acids, such as rumenic and vaccenic acids, which can provide numerous benefits to human health (Dilzer and Park, 2012).

Several factors can affect the chemical composition and FA profile of beef cattle meat (Reddy et al., 2015), including nutrition (Prado et al., 2008a), finishing systems (Aricetti et al., 2008; Padre et al., 2006), and sex (Padre et al., 2007). Cafferky et al. (2019) reported that sex could be a factor that contributes to changes in muscle characteristics because it affects muscle growth and fat deposition in the carcass.

Nutrition is considered a factor that can influence meat quality. However, few studies (Prado et al., 2011; Rotta et al., 2009) have evaluated the relationship between the effect of sex and high-grain diets on the meat quality of animals.

High-grain diets improve animal performance and carcass characteristics, thus obtaining herds that are more homogeneous.

Due to the great number of variables, reaching a reasonable conclusion about meat quality using classical analytical methods is very laborious. Hence, a decrease in the number of quality variables used for certain statistical techniques and analysis is required (Destefanis et al., 2000). Principal component analysis (PCA) is a useful device to evaluate the variations among the meat characteristics and can adequately identify the most important directions of variability in a multivariate data matrix, hence allowing results that can be displayed graphically (Mwove et al., 2018).

Our hypothesis was that the fatty acid profile of meat from cattle fed with high-grain diets would be similar between sexual classes. This study aimed to evaluate the effect of sex on the chemical composition, fatty acid profile, and nutritional indexes of the *Longissimus lumborum* (*L. lumborum*) muscle of feedlot cattle fed a whole shelled corn diet.

## 2 Material and methods

### 2.1 Location and ethical considerations

The experiment was carried out at the Manaus farm located in Itajú do Colônia, BA, Brazil. This study was approved by the Committee of Ethics in Animal Research of the State University of Southwest Bahia (protocol no. 141-2016) and conducted by following the guiding principles of biomedical research with animals of the National Research Council (NRC; 1985).

### 2.2 Animals and experimental design

There were 40 Nellore cattle identified, vaccinated against rabies, dewormed (2.25 % ivermectin + 1.25 % abamectin), and allocated to individual stalls equipped with individual automatic drinkers and feeding. A total of 20 uncastrated bulls with an average body weight of 411 ± 20.82 kg and 30 months old and 20 heifers weighing 274 ± 17.80 kg and aged 24 months old were used in the experiment.

The animals were kept in a feedlot system for 80 d, preceded by 20 d of adaptation (adaptation period) and 60 d for data collection (experimental period). During the adaptation period, the animals received *Brachiaria decumbens* as roughage (ad libitum) and increasing proportions of the whole shelled corn diet. In the experimental period, the animals received a roughage-free, full shelled corn diet. At the end of this period, the average final body weight was 491 ± 20.82 kg for bulls and 343 ± 17.80 kg for heifers.

The diet was composed of 85 % whole shelled corn and 15 % protein–vitamin–mineral supplement (monocalcium phosphate, calcium carbonate, soybean hull, soybean meal, calcium sulfate, animal urea, monensin sodium, and a vitamin and mineral premix) and was offered ad libitum, twice daily, at 08:00 and 16:00 LT (local time). The supplied diets were weighed daily and adjusted to allow for 10 % refusals (Table 1).

**Table 1:** Ratio of ingredients and chemical composition (%; dry matter, DM, basis) of the diet.

Ratio of ingredients	
Whole shelled corn	85.00
Protein–mineral–vitamin supplement${}^{a}$	15.00
Chemical composition	
Dry matter	87.40
Organic matter	83.12
Crude protein	11.06
Ash	4.28
Neutral detergent fiber adjusted to ash and protein	15.06
Acid detergent fiber	10.10
Total carbohydrates	80.81
Non-fiber carbohydrates	65.75
Ether extract	3.85
Total digestible nutrients	74.41
Fatty acids (milligrams per 100 g of FAME)	
Myristic (14 : 0)	<0.01
Palmitic (16 : 0)	15.91
Stearic (18 : 0)	2.09
Oleic and vaccenic (18 : 1 *n*-9)	23.80
Linoleic (18 : 2 *n*-6)	52.80
Linolenic (18 : 3 *n*-3)	1.30
Arachidic (20 : 4 *n*-6)	0.06
Eicosatrienoic (20 : 0)	0.02
Arachidonic (20 : 4 *n*-6)	1.06
Eicosapentaenoic (20 : 5 *n*-3)	0.02
Docosahexaenoic (22 : 6 *n*-3)	0.03
Others and unknowns${}^{b}$	2.91

${}^{a}$ Premix composition per kilogram of supplement includes sulfur 4.5 g, magnesium 0.7 g, potassium 2.7 g, sodium 9.7 g, cobalt 5 mg, copper 175 mg, chromium 1.4 mg, fluorine 130 mg, iodine 5 mg, manganese 182 mg, molybdenum 0.35 mg, nickel 0.3 mg, selenium 1.8 mg, zinc 421 mg, vitamin A 21 000 ui, vitamin D 3000 ui, and vitamin E 140 ui. Guaranteed levels of the supplement are phosphorus 1 %, calcium 4.3 %, ether extract 1.4 %, fiber matter 6.9 %, ash 21.4, crude protein 38.6 %, TDNs (total digestible nutrients) 60 %, NPN (nonprotein nitrogen) 42 %, moisture 10 %, and monensin sodium 150 mg kg${}^{-1}$. ${}^{b}$ Denotes fatty acids identified but not reported or quantified.

Samples of supplied diets and refusals were collected daily and stored for later analysis. Dry matter (DM method 930.15), crude protein (crude protein Kjeldahl (CP-Kjeldahl) procedure; method 976.05), ether extract (EE method 920.39), and ash (method 942.05) contents were determined according to Association of Official Analytical Chemists methods (AOAC, 1990). Neutral detergent fiber (NDF) and acid detergent fiber (ADF) were determined according to the Van Soest et al. (1991) method and corrected for ash and protein (NDFap).

The total carbohydrates (TCs) and nonfibrous carbohydrates (NFCs) were estimated according to Sniffen et al. (1992). The concentration of nonfibrous carbohydrates was determined by the difference between TCs and NDFap. The total digestible nutrients estimated (TDNest) was calculated using the equation described by Cappelle et al. (2001), where TDNest = 91.0246 and 0.571588 NDF.

### 2.3 Slaughter and sample collection

At the end of the experimental period, the animals were weighed after 16 h of solid fasting. Animals were slaughtered at a commercial slaughterhouse, according to the industrial practices in Brazil. Prior to the slaughtering procedures, the animals were stunned by electronarcosis. The carcasses were suspended and bled from the jugular vein and carotid artery before being skinned and eviscerated. Immediately, the carcasses were identified, divided, and weighed to determine the hot carcass yield and were then placed in a cold chamber (4 ${}^{\circ }$C) for 24 h. The loin (*L. lumborum* muscle) from the right side of the carcasses was stored at -24 ${}^{\circ }$C for further analysis.

### 2.4 Chemical composition and fatty acid profile analysis

The chemical composition of the meat was determined following the methods of the AOAC (1990) for DM, CP, and ash.

The fatty acid profiles were obtained from the *L. lumborum* samples using the method of synthesis of fatty acid methyl esters (FAME) described by O'Fallon et al. (2007).

Fresh meat samples were homogenized by grinding them for 10–15 s in a coffee grinder at room temperature; immediately thereafter, the samples were dried by lyophilization for 5 d. The dried meat samples were uniformed by grinding them for 10–15 s in a coffee grinder at 16 ${}^{\circ }$C temperature. To avoid heating up the machine, we waited 1 to 3 min between samples. A total of 0.5 g of the lyophilized sample was placed in a Pyrex culture tube with a screw cap (16 × 125 mm) to which 1.0 mL of the internal standard C19 : 0 (189-19; Sigma-Aldrich; 10 mg of C19 : 0 mL${}^{-1}$ MeOH), 0.7 mL of 10N KOH, and 5.3 mL MeOH was added.

The tube was incubated in a 55 ${}^{\circ }$C water bath for 1.5 h, with vigorous shaking by hand for 5 s every 20 min to properly permeate, dissolve, and hydrolyze the sample. After cooling below room temperature in a cold, tap water bath, 0.58 mL of 24N H${}_{2}$SO${}_{4}$ was added. The tube was mixed by inversion and, with precipitated K${}_{2}$SO${}_{4}$ present, was incubated again in a 55 ${}^{\circ }$C water bath for 1.5 h with shaking by hand for 5 s every 20 min. After FAME synthesis, the tube was cooled in a cold, tap water bath. Then 3 mL of hexane was added, and the tube was vortex-mixed for 5 min in a multi-tube vortex. The tube was centrifuged for 5 min in a tabletop centrifuge, and the hexane layer, containing the FAME, was placed into a gas chromatography (GC) vial. The vial was capped and placed at -20 ${}^{\circ }$C until GC analysis.

The fatty acid composition of the FAME sample was determined by capillary GC on a SPTM-2560, with 100 m × 25 mm × 0.2 µm film thickness (Supelco), installed on a FOCUS GC gas chromatograph equipped with a flame ionization detector and split injection (Thermo Fisher Scientific Inc.). Hydrogenous gas (H${}_{2}$) was used as a carrier gas (1 mL min${}^{-1}$) and nitrogen as an auxiliary gas. Detector and injector temperatures were set at 250 ${}^{\circ }$C, with a split ratio 15 : 1. The oven temperature was set to 70 ${}^{\circ }$C for 4 min and increased by 13 ${}^{\circ }$C min${}^{-1}$ to 175 ${}^{\circ }$C. This temperature was held for 27 min, then increased by 4 ${}^{\circ }$C min${}^{-1}$ to 215 ${}^{\circ }$C, and held for a further 31 min (Kramer et al., 1997). Fatty acids were identified by comparing their retention times with the fatty acid methyl standard described previously.

Atherogenicity (AI) and thrombogenicity (TI) indexes (Ulbricht and Southgate, 1991) and the hypocholesterolemic : hypercholesterolemic (h : H) fatty acid ratio (Santos-Silva et al., 2002) were calculated. Desirable fatty acids (DFAs) were obtained according to Rhee (1992). The activity indexes of the elongase and Δ-9-desaturase enzymes were determined using the methodology proposed by Malau-Aduli et al. (1997) and Kazala et al. (1999), respectively.

### 2.5 Statistical analysis

The chemical composition and FA profile of *L. lumborum* were analyzed for variance using the F test at a 0.05 level of significance, according to the following model: Yijk = μ+ Ti + eijk, where Yijk is the observed value of the dependent variable, μ is the overall mean, Ti is the effect of sex, and eijk represents residual error.

The values of the parameters obtained from the FA profile analysis in cattle fed a whole shelled corn diet produced a multivariate data set and were arranged in a matrix and interpreted using PCA. The analysis was performed using the Statistical Analysis System 9.0 (SAS, 2009) software with a data-centric average.

## 3 Results

The sex of the animals did not influence the chemical composition of the *L. lumborum* muscle. The moisture, ash, total lipid, and crude protein contents did not show differences (p>0.05) between the treatments (Table 2).

**Table 2:** Chemical composition (grams per 100 g of muscle) of *Longissimus lumborum* muscle of feedlot cattle finished with a whole shelled corn diet. Note that SEM is the standard error of the mean.

Item	Sex		SEM	p value
	Bulls	Heifers		
Moisture	74.18 ± 0.41	74.00 ± 0.20	0.154	0.224
Total lipids	4.17 ± 0.11	4.22 ± 0.06	0.044	0.269
Ash	1.78 ± 0.13	1.65 ± 0.20	0.081	0.439
Crude protein	20.02 ± 0.31	19.63 ± 0.22	0.127	0.316

Mean ± standard deviation; p values were considered significant at 0.05.

For the FA composition, the 14 : 0, 15 : 0, and 16 : 0 SFA content was similar between sex groups (p>0.05), whereas 17 : 0, 18 : 0, and 21 : 0 FA content differed (p<0.05) between sexes (Table 3). The concentrations of 16 : 1 *cis*-9, 17 : 1 *cis*-10, and 18 : 1 (*cis*-9 and *trans*-11) were higher (p<0.05) in the *L. lumborum* muscle of heifers. The fatty acids 14 : 1 *cis*-9, 15 : 1 *cis*-10, 22 : 1 *n*-9, and 24 : 1 *n*-9 monounsaturated fatty acids (MUFAs) and the 18 : 3 *n*-3, 20 : 4 *n*-6, and 20 : 5 *n*-3 polyunsaturated fatty acids (PUFAs) did not show differences (p>0.05) between the sexes, whereas the 18 : 2 *cis*-9 and *cis*-12, 18 : 2 *cis*-9 and *trans*-11, 20 : 3 *n*-6, and 22 : 6 *n*-3 PUFAs did present differences (p<0.05) between sexes (Table 3).

**Table 3:** Fatty acid composition (grams per 100 g FAME) of the *Longissimus lumborum* muscle from feedlot cattle finished with a whole shelled corn diet.

Fatty acid		Sex		SEM	p value
		Bulls	Heifers		
Saturated fatty acids (SFAs)					
14 : 0	Myristic acid	3.28 ± 0.19	3.16 ± 0.24	0.104	0.698
15 : 0	Pentadecylic acid	0.52 ± 0.02	0.53 ± 0.03	0.014	0.684
16 : 0	Palmitic acid	28.49 ± 1.25	26.22 ± 0.68	0.479	0.132
17 : 0	Margaric acid	1.60 ± 0.06	1.32 ± 0.04	0.025	<0.001
18 : 0	Stearic acid	15.56 ± 0.46	12.72 ± 0.41	0.208	<0.001
21 : 0	Heneicosanoic acid	0.11 ± 0.01	0.90 ± 0.01	0.019	0.048
Monounsaturated fatty acids (MUFAs)					
14 : 1 *cis*-9	Myristoleic acid	1.05 ± 0.06	0.96 ± 0.07	0.030	0.330
15 : 1 *cis*-10	Pentadecenoic acid	0.13 ± 0.01	0.14 ± 0.01	0.003	0.244
16 : 1 *cis*-9	Palmitoleic acid	3.07 ± 0.14	3.66 ± 0.14	0.067	0.010
17 : 1 *cis*-10	Heptadecenoic acid	0.95 ± 0.03	1.19 ± 0.03	0.014	<0.001
18 : 1 *cis*-9 and *trans*-11	Vaccenic and oleic acids	35.19 ± 0.59	39.69 ± 0.71	0.308	<0.001
22 : 1 *n*-9	Erucic acid	1.37 ± 0.03	1.42 ± 0.03	0.038	0.108
24 : 1 *n*-9	Nervonic acid	0.48 ± 0.02	0.53 ± 0.03	0.011	0.087
Polyunsaturated fatty acids (PUFAs)					
18 : 2 *cis*-9 *cis*-12	Linoleic acid	8.06 ± 0.26	6.05 ± 0.38	0.154	<0.001
18 : 2 *cis*-9 *trans*-11	Conjugated linoleic acid	0.52 ± 0.02	0.34 ± 0.03	0.011	<0.001
18 : 3 *n*-3	α-linolenic acid	0.33 ± 0.02	0.29 ± 0.01	0.008	0.160
20 : 3 *n*-6	Dihomo-γ-linolenic acid	0.89 ± 0.01	0.87 ± 0.01	0.020	<0.001
20 : 4 *n*-6	Arachidonic acid	0.25 ± 0.01	0.27 ± 0.02	0.007	0.479
20 : 5 *n*-3	Eicosapentaenoic acid	0.15 ± 0.01	0.14 ± 0.01	0.005	0.644
22 : 6 *n*-3	Docosahexaenoic acid	0.49 ± 0.01	0.27 ± 0.01	0.019	0.002

Mean ± standard deviation; p values were considered significant at 0.05.

There were differences between sexes (p<0.05) for total SFAs, MUFAs, PUFAs, and omega 6 (*n*-6) FAs (Table 4). No difference was observed for the total omega 3 (*n*-3) FAs (p>0.05). Indexes of nutritional quality, such as the TI, and the *n*-6 : *n*-3 ratio were different between the sexes (p<0.05). No significant differences (p>0.05) in the PUFA : SFA ratio, AI, or h : H ratio were observed between the sexes. The average AI values were 0.84 and 0.73, and the average TI values were 1.76 and 1.45 for bulls and heifers, respectively.

**Table 4:** Total and nutritional quality indexes and desaturase indexes of the *Longissimus lumborum* muscle of feedlot cattle finished with a whole shelled corn diet.

Item	Sex		SEM	p value
	Bulls	Heifers		
Total (milligrams per 100 g FAME)				
Saturated fatty acids	48.13 ± 0.64	44.04 ± 0.97	0.388	0.003
Monounsaturated fatty acids	41.35 ± 0.54	46.18 ± 0.64	0.281	<0.001
Polyunsaturated fatty acids	9.38 ± 0.38	7.57 ± 0.41	0.187	0.005
*n*-6 fatty acids	8.24 ± 0.25	6.28 ± 0.25	0.118	<0.001
*n*-3 fatty acids	0.89 ± 0.04	0.87 ± 0.04	0.020	0.826
PUFA : SFA	0.19 ± 0.01	0.17 ± 0.01	0.005	0.285
*n*-6 : *n*-3	9.07 ± 0.44	7.34 ± 0.46	0.214	0.015
Atherogenicity index	0.84 ± 0.26	0.73 ± 0.15	0.028	0.203
Thrombogenicity index	1.76 ± 0.18	1.45 ± 0.10	0.039	0.020
h : H	1.43 ± 0.22	1.60 ± 0.13	0.042	0.193
Δ-9-desaturase C14 : 0	0.25 ± 0.19	0.23 ± 0.09	0.006	0.024
Δ-9-desaturase C16 : 0	0.10 ± 0.17	0.12 ± 0.09	0.002	0.002
Δ-9-desaturase C18 : 0	0.69 ± 0.05	0.76 ± 0.02	0.005	<0.001

Mean ± standard deviation; p values were considered significant at 0.05. PUFA : SFA is the polyunsaturated : saturated fatty acids ratio, *n*-6 : *n*-3 is the omega 6 : omega 3 fatty acids ratio, h : H is the hypocholesterolemic : hypercholesterolemic fatty acids ratio, and Δ-9-desaturase is the activity index of Δ-9-desaturase.

The principal component analysis showed that 82.63 % of the variation in the results was explained by the first (69.52 %) and second (13.11 %) principal components (PCs; Table 5). These numbers represent the significant contributions of individual FA variables to the total variability explained by the generated PCs. To evaluate the discrimination of the FA profile, the two main components (PC1 and PC2) were plotted (Fig. 1). The score portion for PC1 compared to PC2, with a cumulative contribution of 82.63 %, was the easiest method to view the main trends defined in the different treatment samples.

**Figure 1:**
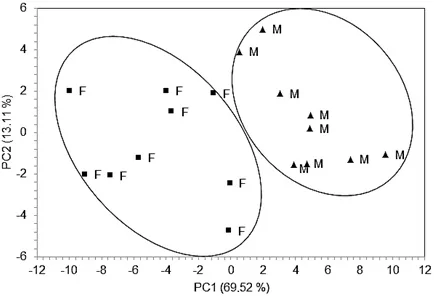
Principal component analysis (PCA) plots for Nellore beef cattle. PCA plots were generated from the FA profile considering cattle sex, i.e., bulls (M) and heifers (F). PC1 and PC2 represent principal components 1 and 2, respectively.

**Table 5:** Principal component (PC), eigenvalues ($\lambda_{i}$), and percentage of variance explained by the component (% VCP) of the fatty acid profile in the *Longissimus lumborum* muscle.

i	Principal	$\lambda_{i}$	% VCP	% VCP
	component			(accumulated)
1	PC1	31.923	69.52	69.52
2	PC2	6.019	13.11	82.63
3	PC3	4.849	10.56	93.19
4	PC4	1.225	2.67	95.86
5	PC5	0.690	1.50	97.36
6	PC6	0.599	1.31	98.67
7	PC7	0.279	0.61	99.28
8	PC8	0.185	0.40	99.68
–	–	–	–	–
–	–	–	–	–
–	–	–	–	–
28	PC28	0.000	0.00	100.00

## 4 Discussion

### 4.1 Chemical composition of the meat

Moisture in the *Longissimus* muscle of bovines finished in feedlot varies from 71.4 % (cull cows) to 75.0 % (bulls); this variation is related to the total lipid content in the muscle (Rotta et al., 2009). In the present study, the moisture content was similar between the sexual classes, and the average between these (74.09 %) is within the parameter found in the literature.

The protein content in muscle is higher in bulls than in heifers (Rotta et al., 2009); however, sex was not observed to affect the protein content of the muscle. Prado et al. (2008a) found a value of 20.41 g/100 g in muscle, similar to the average value of 19.82 g/100 g in muscle obtained in the present experiment.

According to Rotta et al. (2009), sex is a factor that affects ash content. Rotta et al. (2009) found that ash values in the *Longissimus* muscle varied from 1.06 % (bulls) to 1.40 % (cull cows) between sexes, similar to the values found in this study (1.72 %). Paulino et al. (2009) state that factors such as the genetic group, age, sex, diet, and environmental factors influence the patterns of fat deposition in tissues. The average value of total lipids of 4.44 g/100 g in muscle obtained from the total lipid content in the *L. lumborum* muscle of feedlot cattle fed with a whole shelled corn diet is similar to the range of 0.91–7.10 g/100 g in muscle obtained by Rotta et al. (2009). According to Park et al. (2018), the total lipid content in beef cattle meat is influenced by several factors, principally sex and age, because the fat deposition in heifers is premature compared with bulls. Furthermore, testosterone production in males is superior to that in females, which promotes higher muscle growth capacity and reduces the fat deposition capacity in males (Rotta et al., 2009).

Despite the differences observed between sexual classes in relation to fat deposition, these differences vary with the breed of cattle and are more relevant in *Boss taurus* cattle than in *Boss indicus* (Blanco et al., 2020; King et al., 2006). There are very few studies that compare the chemical composition of the meat of the sexual classes of *Boss indicus*. However, when the *Boss indicus* breeds were included in the study, then the chemical composition of the meat did not show differences unless these were influenced by some dietary aspect (Prado et al., 2008b).

The chemical composition of the animals evaluated in the present study was similar to that of Silva et al. (2021) and Gebremariam (2022) for *Boss indicus* cattle.

### 4.2 Fatty acid profile and quality of the meat

Zembayashi et al. (1995) suggested that carcass fat or animal age did not promote the differences in fatty acid composition between sexes; therefore, those differences are related to the ingestive behavior of the animal or to digestive and physiological metabolism. The authors cited that the lipid metabolism in the adipose tissue could be manipulated by the sex hormone status, thus affecting the enzymatic systems influenced by these hormonal changes and promoting differences in the fatty acid composition. The excess energy from ruminant feed is metabolized and stored in the form of adipose tissue, resulting in a higher fat content in the carcass (Chilliard, 1993). The enzymes responsible for FA synthesis and hypertrophy of adipocytes are regulated by the hormone status and the end products of ruminal fermentation, which are determined by the diet composition.

Among the SFAs, 16 : 0 was found at the highest concentration in both sexes, with 28.49 % and 26.22 % in bulls and heifers, respectively, followed by stearic acid (18 : 0) at 15.56 % and 12.72 % (Table 3). Few variations between these two FAs have been reported in the literature, even when comparing feedlot and grazing systems, different diets, genetic groups, or sexes (Fernandes et al., 2009; Prado et al., 2011; Maniaci et al., 2020; Krusinski et al., 2022; Schumacher et al., 2022).

According to Cao et al. (2008), palmitoleic (16 : 1 *n*-7) acid has been proposed to be a lipokine, which is a molecule produced by adipocytes to signal various organs and regulate metabolic homeostasis by stimulating insulin action in muscles and suppressing hepatic steatosis. The diet offered to the cattle in this study did not contain 16 : 1 *n*-7 acid; however, this FA was present in the muscle tissue. A possible explanation for the origin of this FA is lipogenesis, an important metabolic process in ruminants.

The intake of SFAs is related to an increase in cholesterol and low-density lipoprotein (LDL) serum levels, which can result in cardiovascular problems and have negative effects on human health. However, 18 : 1 *cis*-9 FAs and PUFAs have been observed to reduce cholesterol while increasing high-density lipoprotein (HDL) serum levels (Hamułka et al., 2018). Vaccenic acid is considered a nonessential FA and is recognized for its hypocholesterolemic effect (Fernandes et al., 2009), but stearic acid (18 : 0) has not been shown to affect cholesterol serum levels and thus would not result in cardiovascular problems in humans. Therefore, approximately 70 % of the FAs in the *L. lumborum* are in the form of 18 : 0 FAs, MUFAs, or PUFAs, and these fat sources are not linked to heart disease in humans.

Higher concentrations of 18 : 2 *n*-6, 18 : 2 *cis*-9 *trans*-11, and 22 : 6 *n*-3 PUFAs were observed in bulls; of these, the 18 : 2 *n*-6 was found at the highest concentrations. The 18 : 2 *n*-6 fatty acid has a dietary origin, indicating that the differences found in this experiment are related to dietary intake (higher in bulls than heifers), absorption, and nutrient use (Malau-Aduli et al., 2000).

The 18 : 2 *cis*-9 and *trans*-11 FA, the most abundant isomer of conjugated linoleic acid (CLA) in beef cattle muscle, which is most commonly found in beef cattle meat, was present at a concentration of 0.52 % in bulls and 0.34 % in heifers. According to Scollan et al. (2017), the concentration of CLA in beef meat is between 0.34 % and 0.82 %. There is evidence linking this isomer to human health benefits, and therefore, it has been studied in animal products. Although the results are not conclusive, a review of the CLA effects on human health (den Hartigh, 2019) showed that this metabolite exhibited anti-carcinogenic, anti-obesogenic, and anti-atherosclerotic tendencies; however, it cannot protect against type-2 diabetes.

The presence of this FA in ruminant products is a consequence of two processes, namely partial biohydrogenation of dietary FAs (linoleic and linolenic acids) or endogenous desaturation by the Δ-9-desaturase and Δ-5-desaturase enzyme of the trans-vaccenic FA (Ribeiro et al., 2016).

There is evidence showing that the Δ-5-desaturase enzyme has a higher activity or continues its activity for longer periods in the muscle tissue of bulls than heifers (Malau-Aduli et al., 2000), as it is modulated by the hormonal status (Dias et al., 2018), thus explaining the lower vaccenic and oleic acids values in bulls. Therefore, the lower values of vaccenic and oleic acids in bulls indicate a contribution for the CLA content (tissue metabolism). In this sense, it is possible to infer that vaccenic and oleic acids were desaturated to CLA, and this effect was greater in bulls. For this reason, the content of this FA is lower, and the content of CLA is higher in this sexual class.

According to Reddy et al. (2015), the contents of C18 : 1 (oleic acid) and MUFAs are higher in heifers and C16 : 0 (palmitic acid), C18 : 0 (Stearic acid), and saturated fatty acids (SFAs) are higher in bulls and steers than heifers and cows. This statement corroborates the results obtained in the present study. Furthermore, Silva et al. (2021), Blanco et al. (2020), Sobczuk-Szul et al. (2021), and Nogalski et al. (2018) found similar contents and proportions between sex classes and within the fatty acid profile.

Besides, the Δ-9-desaturase C14, Δ-9-desaturase C16, and Δ-9-desaturase C18 desaturase indexes presented significant differences between sexes (p<0.05; Table 4). Endogenous synthesis of CLA involves Δ-9-desaturase activity and desaturation of linoleic acid, resulting in C18 : 1 *trans*-11 from C18 : 0 (Corl et al., 2001).

In this study, heifers presented higher desaturase indexes than bulls, explaining the higher MUFA content in heifers. These results are in accordance with Fernandes et al. (2009), in which the authors indicate that higher indexes may be related to increased activity of the Δ-9-desaturase enzyme in heifer muscles; this enzyme plays a role in the conversion of myristic, palmitic, and stearic acids to their corresponding monounsaturated acids (Kazala et al., 1999).

The higher PUFA content, as observed in bulls, is explained by the higher activity of desaturase enzyme in bulls than heifers, considering that this enzyme is responsible for the synthesis of PUFA from MUFA in the muscle (Malau-Aduli et al., 2000). This observation is corroborated by the lower MUFA values in bulls, when compared to heifers, supporting the higher PUFA values in each sexual class as a result of desaturation.

Another important characteristic to remember regarding PUFAs is that the *n*-6 : *n*-3 ratio represents the balance of this FA family, which determines the effectiveness of the essential FAs present in the phospholipids that make up cell membranes. Alothman et al. (2019) observed that meat from pasture-fed animals has a lower *n*-6 : *n*-3 ratio than meat from animals that consume grain because pasture has higher levels of α-linolenic acid.

The average AI values were 0.84 and 0.73, and the average TI values were 1.76 and 1.45 for bulls and heifers, respectively. These indexes are related to pro- and anti-atherogenic acids and indicate the potential for stimulating platelet aggregation. The smaller values for AI and TI indicate a higher proportion of anti-atherogenic FAs in fat and, consequently, a greater potential for the prevention of heart disease. The h : H ratio is based on the functional effects of FAs on cholesterol metabolism; however, this ratio also allows for nutritional assessment, considering the beneficial effects of the MUFAs described in this ratio.

We investigated the similarities in the FA profile of cattle meat to better understand the relationships between variables. The contributions of the first (69.52 %) and second (13.11 %) principal components represent the significant contributions of individual FA to the total variability. There was a tendency for separation of the samples in accordance with sex in the FA profile of the *L. lumborum* muscle of feedlot cattle fed a whole shelled corn diet. The score portion for PC1 compared to PC2, with a cumulative contribution of 82.63 %, was the easiest method to view the main trends defined in the different treatment samples. According to Laureati et al. (2014), multivariate statistical techniques, such as PCA, can be used to evaluate meat quality, with the aim of correlating variables and through simple linear combination of original variables. The most important variables can be identified and will be the first principal components (Ferreira et al., 2000).

## 5 Conclusion

Bulls and heifers fed a whole shelled corn diet and finished in a feedlot system mainly showed differences in the fatty acid profile of the meat, indicating that the fatty acid profile in the current study was more influenced by gender than by diet. In this sense, uncastrated bulls showed higher proportions of saturated fatty acids than heifers. However, bulls have the highest *n*-6 FA content and *n*-6 : *n*-3 ratio and more anti-atherogenic and anti-thrombogenic FAs, which are beneficial to human health. On the other hand, feedlot heifers fed the same diet had a higher concentration of palmitoleic (16 : 1 *n*-7), heptadecenoic (17 : 1 *n*-7), oleic (18 : 1 *cis*-9), and vaccenic (18 : 1 *trans*-11) acids, which are MUFAs recommended for human consumption.

## Data Availability

The original data used in this study are available from the corresponding author upon request.
